# An Unusual Case of Anuric Acute Kidney Injury Secondary to the Use of Low-Dose Acetazolamide as Preventive Management for Acute Mountain Sickness

**DOI:** 10.3390/diseases13070228

**Published:** 2025-07-21

**Authors:** Marco Dominguez Davalos, Raúl Valenzuela Córdova, Celia Rodríguez Tudero, Elena Jiménez Mayor, Carlos Bedia Castillo, José C. De La Flor, Roger Leon Montesinos, Cristian León Rabanal, Michael Cieza Terrones, Javier A. Neyra

**Affiliations:** 1 Department of Nephrology, Hospital Cayetano Heredia, Lima 15002, Peru; marco.dominguez.d@upch.pe (M.D.D.); carlos.valenzuela@upch.pe (R.V.C.); cristian.leon.r@upch.pe (C.L.R.); 2 Faculty of Medicine, Peruana Cayetano Heredia University, Lima 15002, Peru; carlos.bedia@upch.pe; 3 Department of Nephrology, Hospital Universitario de Salamanca, 37007 Salamanca, Spain; 4 Surgery Department, Faculty of Medicine, University of Salamanca, 37007 Salamanca, Spain; 5 Department of Nephrology, Hospital San Pedro de Alcántara, 10001 Cáceres, Spain; elena.jimenezm@salud-juntaex.es; 6 Department of Rheumatology, Hospital Cayetano Heredia, Lima 15002, Peru; 7 Department of Nephrology, Hospital Central Defense Gomez Ulla, 28047 Madrid, Spain; jflomer@mde.es; 8 Health Sciences Doctoral Program, Faculty of Medicine, Alcala University, 28805 Madrid, Spain; 9 Department of Medicine and Medical Specialties, Faculty of Medicine, Alcala University, 28805 Madrid, Spain; 10 Department of Nephrology, University Clinical Hospital, La Paz 0201, Bolivia; rleonm@univalle.edu; 11 Department of Engineering, Faculty of Science and Engineering, Peruana Cayetano Heredia University, Lima 15002, Peru; michael.cieza@upch.pe; 12 Department of Internal Medicine, Division of Nephrology, Bone and Mineral Metabolism, University of Kentucky, Lexington, KY 40508, USA; jneyra@uabmc.edu; 13 Charles and Jane Park Center for Mineral Metabolism and Clinical Research, University of Texas Southwestern Medical Center, Dallas, TX 75080, USA; 14 Department of Internal Medicine, Division of Nephrology, University of Alabama at Birmingham, Birmingham, AL 35250, USA

**Keywords:** acute kidney injury, acetazolamide, crystalluria, altitude sickness/drug therapy, anuria

## Abstract

**Background/Objectives**: Acetazolamide is widely used for acute mountain sickness (AMS) prophylaxis. Whilst generally safe, acute kidney injury (AKI) is a rare but serious adverse event. We present a case of anuric AKI following minimal exposure to acetazolamide, contributing to the limited literature on its nephrotoxicity at prophylactic doses. **Methods**: A 54-year-old previously healthy male ingested 250 mg/day of oral acetazolamide for two days. He developed acute anuria and lumbar pain. Diagnostic evaluation included laboratory tests, imaging, microbiological cultures, autoimmune panels, and diuretic response. No signs of infection, urinary tract obstruction, or systemic disease were found. **Results**: The patient met KDIGO 2012 criteria for stage 3 AKI, with peak serum creatinine of 10.6 mg/dL and metabolic acidosis. Imaging confirmed non-obstructive nephrolithiasis. Conservative treatment failed; intermittent hemodialysis was initiated. Renal function recovered rapidly, with the normalization of serum creatinine and urinary output by day 4. **Conclusions**: This case represents the lowest cumulative dose of acetazolamide reported to cause stage 3 AKI. The findings support a pathophysiological mechanism involving sulfonamide-induced crystalluria and intratubular obstruction. Physicians should consider acetazolamide in the differential diagnosis of AKI, even with short-term prophylactic use.

## 1. Introduction

Acetazolamide (ACZ) is a sulfonamide-derived drug that acts as a non-competitive inhibitor of carbonic anhydrase, primarily in the proximal tubule, increasing renal excretion of bicarbonate, sodium, and water, leading to alkaline diuresis and normal anion gap metabolic acidosis. It is not metabolized and is excreted unchanged in the urine [1].

ACZ is commonly used for the treatment of various clinical conditions, including glaucoma, decompensated heart failure, pulmonary hypertension, pseudotumor cerebri, seizures, metabolic alkalosis, periodic paralysis, hypochloremia, and prophylaxis against altitude sickness [2]. The most common adverse events associated with ACZ use include paresthesia, dysgeusia, nausea, polyuria, and fatigue [3].

Renal complications associated with acetazolamide use occur in the following two distinct forms, with different mechanisms and timelines: [1] acute kidney injury (AKI) due to acute exposure and [2] nephrolithiasis due to long-term use [4]. AKI is a potentially severe but rare complication, with a mechanism which is primarily explained by crystalluria, intratubular obstruction, and retrograde urinary flow [5]. The absence of histological diagnosis in most of the relevant literature is attributed to the rapid recovery of renal function in most cases.

Acute mountain sickness (AMS) affects more than 25% of individuals ascending to 3500 m and over 50% of those reaching altitudes above 6000 m. AMS can progress from nonspecific symptoms to life-threatening high-altitude cerebral edema in less than 1% of cases [6]. The first descriptions of AMS date back to late 19th-century observations by European travelers to the Andes Mountains in South America, notably Edward Whymper [7].

Regarding pathophysiology, during high-altitude ascent susceptible individuals may develop relative hypoventilation and hypoxia, triggering multiple pathological responses that contribute to disease onset. Increased capillary permeability, driven by a complex interaction between fluid mechanics (regional hyperperfusion and elevated hydrostatic pressure) and biohumoral responses (inflammatory mediator release), along with impaired compensatory mechanisms (e.g., reduced ability to mitigate intracranial volume fluctuations), leads to cerebral vasogenic edema [8].

Prophylactic management consists of ACZ 125 mg every 12 h, initiated the day before ascent and continued for two to four days after reaching the target altitude. For AMS treatment, a dose of 250 mg every 12 h is recommended [6].

We present the case of anuric AKI in a man with no significant medical history, following ACZ use as AMS prophylaxis.

## 2. Case Report

A 54-year-old male with no significant medical history ingested ACZ 250 mg/day orally for two doses as prophylaxis for AMS due to a planned trip to the city of Huancavelica, Peru (3676 m above sea level). Twelve hours after the second dose of ACZ, he developed severe right lumbar pain and a reduced urinary output that progressed to anuria. He was subsequently transferred to Lima (at sea level), where anuria and pain persisted. Upon admission, the patient was in fair general condition, with adequate hydration, lucid, and oriented in time, space, and person. His vital signs were as follows: blood pressure (BP) 117/70 mmHg, heart rate (HR) 97 bpm, and oxygen saturation of 97%. He presented with severe low back pain, while the rest of the physical examination was non-contributory. Bladder catheterization confirmed anuria.

Laboratory tests revealed hemoglobin (Hb) 15.8 g/dL, leukocytes 17,600/μL 17.6 × 10^9^/L without left shift, C-reactive protein (CRP) 280 g/dL, serum creatinine (sCr) 5.84 mg/dL, and urea 77 mg/dL. The remaining laboratory results are shown in Table 1.

**Figure 1:**
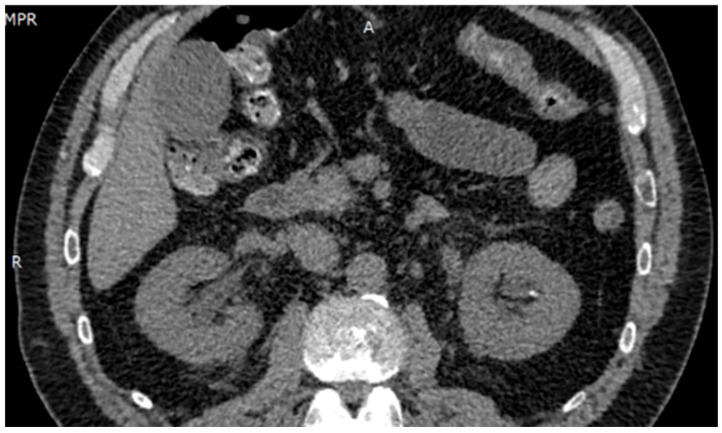
UROTAC showing uncomplicated nephrolithiasis.

Ultrasound imaging showed a 3.5 mm calculus in the lower left calyx, with no evidence of obstruction. Renal Doppler ultrasound revealed no abnormalities. Urotomography (UROTAC) confirmed left renal lithiasis without obstructive effect and an empty bladder (Figure 1).

Negative blood and urine cultures were obtained, along with negative infectious and autoimmune serologies, including hepatitis B surface antigen, hepatitis C antibody, human immunodeficiency virus-1 antibody, venereal disease research laboratory (VDRL) test, complement levels, antinuclear antibody, antineutrophil cytoplasmic antibody, anti-streptolysin O antibody, and rheumatoid factor. Creatine phosphokinase levels were normal.

Urinalysis revealed a pH of 6.5, dipstick 2+ for albumin, specific gravity of 1010, red blood cells > 100/high-power field (HPF), leukocytes 6–8/HPF, sparse epithelial cells, and no crystals or casts. The 24 h proteinuria was 950 mg.

After ruling out obstructive, infectious, and autoimmune etiologies, ACZ ingestion was suspected as the likely cause of AKI, classified as stage 3 according to the Kidney Disease: Improving Global Outcomes (KDIGO) 2012 criteria [9].

Venous excess ultrasound score (VExUS) and lung ultrasound score (LUS) assessments showed no signs of systemic or pulmonary vascular congestion. Consequently, intravenous (IV) fluid therapy with 1500 mL of isotonic saline was initiated, followed by IV administration of 60 mg furosemide to induce diuresis; however, no response was observed. Pain management included IV paracetamol and morphine.

At 24 h post-admission, anuria persisted, with an increase in creatinine and urea to 10.1 mg/dL and 110 mg/dL, respectively. Arterial blood gas analysis revealed an arterial pH of 7.29, PCO_2_ of 26.2 mmHg, HCO_3_^−^ of 13 mmol/L, and anion gap of 23.7 mmol/L, leading to the decision to initiate hemodialysis (HD), which was performed for two consecutive days.

**Figure 2:**
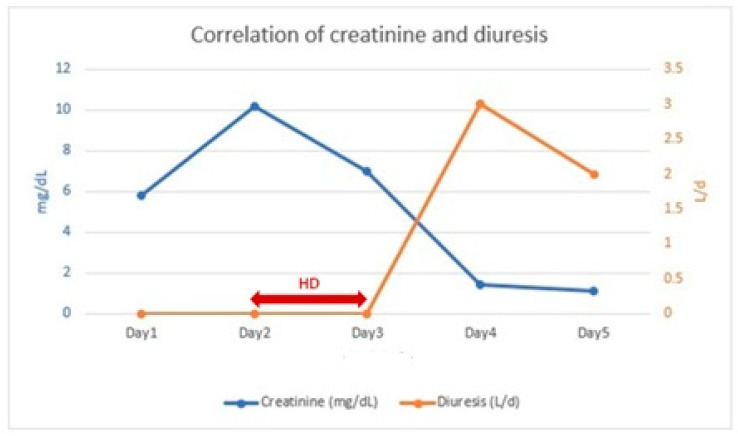
Correlation of creatinine and diuresis.

Within 24 h after the second HD session, the patient exhibited a urine output of 3010 mL/24 h and a decrease in serum creatinine to 1.46 mg/dL (Figure 2). In the following 24 h, renal function continued to improve (sCr: 1.19 mg/dL) with a urine output of 2000 mL/24 h.

On the fifth day post-admission, due to favorable clinical evolution, the patient was discharged. One week later, during outpatient follow-up, laboratory results showed 24 h proteinuria of 180 mg, serum creatinine of 0.8 mg/dL, and a normal urinalysis.

## 3. Discussion

We present an unusual case of anuric AKI in a patient who ingested ACZ as prophylaxis for AMS. AMS is caused by cerebral edema associated with hypobaric hypoxia, which affects individuals who ascend too rapidly to high altitudes beyond their current level of acclimatization. ACZ is recommended as a preventive therapy due to its ability to enhance the poikilocapnic hypoxic ventilatory response, leading to increased partial pressure of arterial oxygen. Additionally, it reduces cerebrospinal fluid production, which may contribute to its beneficial effects [6]. To our knowledge, this is the first reported case of stage 3 AKI, according to KDIGO 2012 criteria [9], associated with the lowest documented cumulative dose of acetazolamide in the literature.

ACZ is a sulfonamide derivative and a carbonic anhydrase inhibitor, primarily acting in the proximal tubule. It is excreted unchanged in the urine, and in acidic urine it forms crystals within the renal tubules. AKI has been recognized as a possible complication of its use; however, this effect remains relatively unknown [2]. In our patient, AKI occurred with a total ACZ dose of 500 mg (250 mg every 24 h for two days), which is even lower than the previously lowest documented cumulative dose in the literature. López-Romero et al. reported a similar case in a 76-year-old man who developed stage 3 anuric AKI (KDIGO 2012) without urological evidence of obstruction after receiving ACZ after cataract surgery (250 mg three times daily for one day) [10].

Most patients with AKI due to acetazolamide present with symptoms such as unilateral or bilateral low back pain, anuria, nausea, and vomiting. Our patient exhibited predominant right lumbar pain and anuria, similar to the case reported by Liu et al., where a 52-year-old man who took ACZ for bilateral intermittent acute angle closure glaucoma symptoms (450 mg per day for 10 days) developed right-sided low back pain and anuria [5].

In our case, the onset of symptoms occurred 36 h after starting ACZ. A similar timeframe was reported by López-Romero et al., who documented symptom onset 24 h after initiating ACZ [10]. However, longer latency periods have been described, such as the case reported by Higenbottam et al. where a 46-year-old patient developed AKI symptoms three weeks after starting ACZ therapy [11].

Notable laboratory findings in our patient were microscopic hematuria and significant proteinuria. López-Menchero et al. reported a case of a 58-year-old man taking ACZ for serous maculopathy (750 mg per day for four days) who developed low back pain and stage 3 anuric AKI without radiological evidence of obstruction or thrombosis. Urine microscopy showed red blood cells but no casts or crystals [12]. Similarly, in the case reported by Neyra et al. microscopic hematuria and proteinuria (240 mg/24 h) were documented, although the latter was not as pronounced as in our case [13].

The majority of patients with AKI due to acetazolamide experience improvement in renal function upon discontinuation of the drug, intravenous volume expansion with 0.9% saline, and, if necessary, sodium bicarbonate (to increase urinary pH), achieving spontaneous resolution and rarely requiring renal replacement therapy (RRT). However, in our patient, despite conservative management, anuria persisted, and azotemia worsened, necessitating two sessions of HD, similar to the cases reported by López-Romero et al. and Neyra et al. Unlike our case, however, these two patients had received higher doses of ACZ—750 mg and 1250 mg, respectively [10,13].

The most conclusive evidence of ACZ-induced AKI was described by Rossert in 1989. Renal biopsy revealed tubular damage with disruption of the tubular basement membrane, cellular debris, and intratubular crystalluria. Tamm-Horsfall protein was detected in Bowman’s space, suggesting intratubular obstruction and retrograde urine flow due to crystal formation [14]. It is noteworthy that the absence of a histopathological diagnosis in most reported cases is attributed to the rapid recovery of renal function.

Acute interstitial nephritis (AIN) due to acetazolamide (ACZ) remains an important differential diagnosis as sulfonamides along with beta-lactam are among the leading causes of drug-induced AIN. Regarding clinical features, back pain may be present in over 30% of cases [15], likely reflecting the edema-induced distension of the renal capsule, and a rapid improvement in renal function is typically observed, as occurred in our patient. The classical triad of fever, rash, and eosinophilia, although absent in this case, is reported in fewer than 10% of patients. A key laboratory finding is C-reactive protein (CRP), which is frequently elevated in AIN. Although the ACZ dose was low, this does not exclude AIN as the allergic reaction is dose-independent.

On the other hand, drug-induced AIN usually develops several days to weeks after exposure to the offending agent, and the clinical course is generally non-oliguric [16]. Therefore, intratubular crystallization remains the most likely diagnosis. Nonetheless, as a renal biopsy was not performed, the presence of AIN cannot be definitively excluded, and it is even possible that both mechanisms contributed to the pathogenesis of the acute kidney injury (AKI).

On the other hand, drug-induced AIN generally develops several days to weeks after exposure to the offending agent, and the clinical course is typically non-oliguric [16]. Therefore, intratubular crystallization remains the most likely diagnosis. However, since a renal biopsy was not performed, AIN cannot be definitively excluded, and a dual mechanism involving both etiologies may have contributed to the development of AKI.

In our case, the development of AKI following exclusive ACZ exposure and the absence of imaging evidence of urinary obstruction strongly supports a pathogenic mechanism involving intratubular obstruction due to sulfonamide crystalluria induced by ACZ. It is important to highlight that crystalluria findings on urinary microscopy are rarely reported in AKI secondary to ACZ [17]. The clinical presentation of anuria and renal colic is characteristic. Urine microscopic examination frequently reveals red blood cells, although sulfonamide crystals have rarely been observed in most cases of ACZ-induced AKI.

Our case is one of the few reported cases of AKI following ACZ prophylaxis for acute mountain sickness. Intravascular volume expansion with isotonic fluids is recommended for renal recovery, which typically occurs within 96 h after ACZ discontinuation. In some cases, by increasing urinary pH, sodium bicarbonate prevents the precipitation of sulfonamide crystals [18]. The importance of considering ACZ as a potentially nephrotoxic agent and including it in the differential diagnosis of anuric AKI is emphasized.

## 4. Conclusions

In conclusion, we describe the clinical and laboratory manifestations of an unusual case of stage 3 anuric AKI following the ingestion of 500 mg of ACZ for acute mountain sickness prophylaxis—a rare adverse effect at such a low cumulative dose, with the possibility of AIN due to a dose-independent allergic reaction. Although intratubular sulfonamide crystallization appears to be the most likely cause, AIN cannot be ruled out in the absence of a renal biopsy. Given the widespread use of ACZ in various medical conditions, physicians should be aware of this potential complication and consider it in the differential diagnosis of patients with ARF and a history of its use.

## Figures and Tables

**Table 1:** Laboratory findings.

	Day 1	Day 2	Day 4	Normal Range-United
Hemoglobin (Hb)	15.8	14.1	14	11–15 g/dL
Platelet count	289	259	257	150–400 × 10^3^ µL
White blood cells (WBCs)	17.6	17.99	8.35	5–10 × 10^3^ µL
Glucose	106		98	75–110 mg/dL
Urea	77.1	110	32.9	15–36 mg/dL
Creatinine	5.84	10.6	1.11	0.7–1.2 mg/dL
Sodium	141.8	140.7		135–148 mmol/L
Chloride	106.5	108.3		98–106 mEq/L
Potassium	4.55	4.66		3.5–5.3 mmol/L
Ionic Calcium		1.14		1.1–1.3 mmol/L
Phosphorus		4.1		2.5–4.5 mg/dL
Magnesium		2.44		1.6–2.3 mg/dL
pH	7.29	7.28	7.42	7.35–7.45
HCO_3_^−^	13	13.1	20.7	21–26 mEq/L
pCO_2_	26.2	27.7	31.1	35–48 mmHg
pO_2_,	86.6	83.4	77.6	83–108 mmHg
pO_2_/FIO_2_	412.2	397.3	371.3	>300 mmHg
CRP	28.03	79.97	50.42	mg/L
Hematuria	0–2 hpf	>100 hpf		<5 hpf
Blood culture		Negative		Negative
Urine culture		Negative		Negative
24 h urine total protein excretion			950	42–225 mg/24 h
ANA		Negative		≤1:80 IIF
Anti-dsDNA		Negative		<30 IU/mL
C3		148		90–180 mg/dL
C4		27		10–40 mg/dL
p-ANCA		Negative		<20 IU/mL
c-ANCA		Negative		<20 IU/mL

NA: Not applicable, pH: hydrogen potential, HCO_3_^−^: bicarbonate, pCO_2_: partial pressure of carbon dioxide, pO_2_: partial pressure of oxygen, pO_2_/FIO_2_: relationship between arterial oxygen and inspired fraction oxygen, CPR: C-reactive protein. Antibody, ANA: antinuclear antibody, anti-dsDNA: antibody against double-stranded DNA, C3: complement C3, C4: complement C4, ANCA: antibody against neutrophils cytoplasm. DH: hospitalization day.

## Data Availability

No new data were created or analyzed in this study. The data used to support the findings of this study are available from the corresponding author on request (contact elena.jimenezm@salud-juntaex.es (E.J.M.)).
